# α-Synuclein Induces the GSK-3-Mediated Phosphorylation and Degradation of NURR1 and Loss of Dopaminergic Hallmarks

**DOI:** 10.1007/s12035-021-02558-9

**Published:** 2021-10-05

**Authors:** Ángel Juan García-Yagüe, Isabel Lastres-Becker, Leonidas Stefanis, Demetrios K. Vassilatis, Antonio Cuadrado

**Affiliations:** 1 Department of Biochemistry, Medical College, Autonomous University of Madrid (UAM), Madrid, Spain; 2 Instituto de Investigaciones Biomédicas “Alberto Sols” (CSIC-UAM), Madrid, Spain; 3 Instituto de Investigación Sanitaria La Paz (IdiPaz), C/ Arturo Duperier, 4, 28029 Madrid, Spain; 4 Centro de Investigación Biomédica en Red de Enfermedades Neurodegenerativas (CIBERNED), Valderrebollo 5, Madrid, Spain; 5 1St Department of Neurology, Aiginition University Hospital, National and Kapodistrian University of Athens, Athens, Greece; 6 National and Kapodistrian University of Athens, Athens, Greece; 7 Center of Clinical Research, Biomedical Research Foundation, Experimental Surgery and Translational Research, Academy of Athens, Athens, Greece

**Keywords:** Parkinson’s disease, Dopaminergic neurons, Transcription, Dopaminergic phenotype

## Abstract

In Parkinson’s disease, the dysfunction of the dopaminergic nigrostriatal tract involves the loss of function of dopaminergic neurons of the substantia nigra pars compacta followed by death of these neurons. The functional recovery of these neurons requires a deep knowledge of the molecules that maintain the dopaminergic phenotype during adulthood and the mechanisms that subvert their activity. Previous studies have shown that transcription factor NURR1, involved in differentiation and maintenance of the dopaminergic phenotype, is downregulated by α-synuclein (α-SYN). In this study, we provide a mechanistic explanation to this finding by connecting α-SYN-induced activation of glycogen synthase kinase-3 (GSK-3) with NURR1 phosphorylation followed by proteasomal degradation. The use of sequential deletion mutants and single point mutants of NURR1 allowed the identification of a domain comprising amino acids 123-PSSPPTPSTPS-134 that is targeted by GSK-3 and leads to subsequent ubiquitination and proteasome degradation. This study provides a detailed analysis of the regulation of NURR1 stability by phosphorylation in synucleinopathies such as Parkinson’s disease.

**Graphical abstract:**

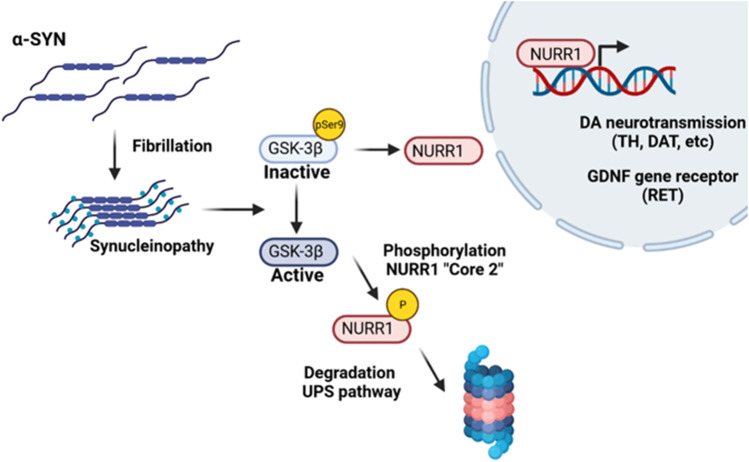

**Supplementary Information:**

The online version contains supplementary material available at 10.1007/s12035-021-02558-9.

## Introduction

Midbrain dopaminergic (DAergic) neurons are the main source of dopamine (DA) in the mammalian central nervous system. Several transcription factors have been implicated in DAergic differentiation [1]. Among them, nuclear receptor‐related factor 1 (NURR1; also known as NR4A2) is a transcription factor of the orphan nuclear receptor class that participates in acquisition of the DAergic phenotype in neurons during development and in the maintenance of their functionality during adulthood [2–4]. It regulates the expression of several genes involved in DA metabolism, including tyrosine hydroxylase (*TH*) [5–7], dopamine transporter (*DAT*) [8], amino acid decarboxylase (*AADC*) [9], vesicular monoamine transporter-2 (*VMAT2*) [9], as well as other non-DAergic genes such as *NRP1* [10] and *RET* (GDNF receptor) [11]. Ablation of NURR1 in adult rodents results in reduced expression of its target genes and loss of functional DAergic midbrain neurons [12–14]. Mutations in the human *NURR1* gene have been identified in association with Parkinson’s disease (PD), where neurodegeneration of the DAergic neurons of the SN occurs [15, 16].

DAergic neuronal loss is associated with abnormal accumulation and aggregation of the protein α-synuclein (α-SYN) in the form of Lewy bodies and Lewy neurites [17]. Point mutations, duplications, and triplications of α-SYN gene (*SNCA*) are associated with familiar forms of PD [18, 19], which indicate a key role of this protein in the neurodegenerative process of the disease. The toxicity elicited by α-SYN oligomers correlates with its phosphorylation at serine 129 as this event promotes fibril formation [20, 21]. Related to NURR1, a seminal study, demonstrated that aberrant expression of human α-SYN in murine DAergic neurons correlates with exacerbated proteasomal degradation of NURR1 and loss of the DAergic phenotype [22]. However, little is known about the molecular mechanisms that connect synucleinopathy with loss of NURR1 stability and loss of DAergic neuron functionality, which may be more important than frank cell loss [3, 12]. This information is crucial to identify early signs of damaged DAergic neurons and to apply a neuroprotective therapy before the manifestation of DAergic cell death.

Phosphorylation is a mechanism used in many proteins to target their proteolytic degradation. For instance, glycogen synthase kinase 3 (GSK-3) phosphorylates several proteins to create a phosphorylation-dependent degradation domain (phosphodegron) that is then recognized by a variety of E3 ubiquitin ligase adapters leading to proteasomal degradation of the phosphorylated protein [23]. Considering that GSK-3 is activated by α-SYN aggregates [24–28], here, we analyzed if NURR1 is phosphorylated by GSK-3 and sent to ubiquitin proteasome degradation phosphorylation by GSK-3. The two isoforms of GSK-3 (GSK-3α and GSK-3β) play critical roles in metabolism, neurogenesis, proliferation, neuronal differentiation, and neuronal death [29], and their dysregulation is associated with neurodegenerative diseases. For instance, abnormal GSK-3β activity leading to TAU phosphorylation and aggregation has been extensively reported in Alzheimer’s disease [30–32] but also in connection with several hallmarks of PD [33–36]. Thus, in postmortem PD brain samples, GSK-3β activity is increased in regions related to PD pathology, and GSK-3β co-localizes with α-SYN in Lewy bodies [35, 36]. α-SYN pathology leads to GSK-3β activation, subsequent phosphorylation of several transcription factors such as Jun, Myc, HSF-1, and CREB, and neuronal death, thus opening the possibility of a similar regulation of NURR1 [28].

In this study, using several models of synucleinopathy, we found that α-SYN-induced activation of GSK-3β leads to phosphorylation of NURR1 and its subsequent ubiquitin–proteasome degradation, which precedes loss of the DAergic phenotype.

## Materials and Methods

### A detailed description of methods is presented in Supplemental Material

#### Cell Culture and Reagents

Human embryonic kidney 293 T with SV40 T antigen (HEK293T) cells were grown in Dulbecco’s modified Eagle’s medium (DMEM) (Sigma-Aldrich, Madrid, Spain) supplemented with 10% fetal bovine serum (Invitrogen, CA, USA) and 80 µg/ml gentamycin (Gibco, MA, USA). Human neuroblastoma cells (SH-SY5Y) were cultured in RPMI supplemented with 10% fetal bovine serum (Invitrogen) and 80 µg/ml gentamicin. The SH-SY5Y-α-SYN Tet-Off cells, described previously [37, 38], were cultured in RPMI with 10% fetal bovine serum, 250 µg/ml G418 (Gibco), 50 µg/ml hygromycin B (Invitrogen), and 2 µg/ml doxycycline (DOX) (Sigma-Aldrich). The expression of α-SYN was switched on by DOX removal. Transient transfection of HEK293T cells was performed with TransFectin Lipid Reagent (Bio-RAD, CA, USA). The inhibitors SB216763, LY294002, and MG132 were from Sigma-Aldrich. Cycloheximide (CHX) was from Boehringer Mannheim (Stuttgart, Germany).

#### Plasmids and Lentiviruses

The vectors pCGN-HA-GSK-3β^Δ9^, pCGN-HA-GSK-3β^WT^, and pCGN-HA-GSK-3β^Y216F^ were provided by Dr. Akira Kikuchi (Department of Biochemistry, Faculty of Medicine, Hiroshima University). Vectors pGL3-NBRE3xLuc and pGL3-TkLuc were provided by Dr. Philippe Lefebvre (INSERM Institut Pasteur de Lille, Lille, France). The HA-Ubiquitin expression vector was provided by Dr. Tadashi Nakagawa (Division of Cell Proliferation, ART, Tohoku University Graduate School of Medicine, Sendai, Japan). The plasmid pcDNA3.1-Nurr1^WT^-V5/6xHis has been described previously [39]. The lentiviral particles used in this study were purchased from Addgene and were generated in HEK293T cells as described previously ([40] and Supplemental Material).

#### α-SYN Pre-formed Fibrils (PFF)

Purified monomeric α‐SYN was purchased from Proteos, Inc (cat no. RP‐003), and PFFs were formed according to the protocol provided by the manufacturer [41, 42] (see Supplemental Material).

#### Immunoblotting

This protocol was performed as described in [43]. Briefly, cells were homogenized in lysis buffer (TRIS pH 7.6 50 mM, 400 mM NaCl, 1 mM EDTA, 1 mM EGTA, and 1% SDS), and samples were heated at 95 °C for 15 min, sonicated and pre-cleared by centrifugation. Proteins were resolved in SDS-PAGE and transferred to Immobilon-P (Merck-Millipore, MA, USA) membranes. Proteins of interest were detected with the primary antibodies indicated in the Supplemental Table 2. Proper peroxidase-conjugated secondary antibodies were used for detection by enhanced chemiluminescence (GE Healthcare).

#### Immunofluorescence

SH-SY5Y cells were seeded in 24-well plates (5 × 10^3^ cells/well) on poly-D-Lys-covered slides and treated with 1 µg/ml PFFs. The protocols have been previously described [39, 44, 45]. Primary antibodies recognized TH (Merck-Millipore), human α-SYN (Santa Cruz Biotechnology, Dallas, TX, USA) and α-SYN-pSer^129^ (Abcam, Cambridge, UK). Secondary antibodies were as follows: Alexa Fluor 488 donkey anti-mouse, and Alexa 546 donkey anti-rabbit (1:500; Thermo Fisher Scientific, Waltham, MA, USA) and Alexa Fluor 546-conjugated donkey anti-mouse IgG (Molecular Probes, Eugene, OR, USA). Control sections were treated identically but omitting the primary antibody.

#### In vivo Ubiquitination Assay

HEK293T cells co-transfected with expression plasmids for HA-Ubiquitin (HA-Ub), Nurr1^WT^-V5/6xHis, or Nurr1^MUT2^-V5/6xHis with pCGN-HA-GSK-3β^Δ9^ or pCGN-HA-GSK-3β^Y216F^, using TransFectin Lipid Reagent (Bio-RAD). After 5 h, HEK293T cells were treated for 16 h with 2 μM MG132 (Sigma-Aldrich). Cells were then lysed in a RIPA buffer (150 mM NaCl, 25 mM Tris–HCl, pH 7.5, 1% Nonidet P-40, 1% sodium deoxycholate, 1% Triton-X100, 0.1% SDS, 1 mM phenylmethylsulfonyl fluoride, 1 mM NaF, 1 mM sodium pyrophosphate, 1 mM sodium orthovanadate, 1 g/ml leupeptin). Then, samples were kept for 30 min at 4 °C in a rotating wheel and centrifuged at 13,000 rpm for 10 min. Three microliters of the anti-V5 (Invitrogen) was added per lysate, and after incubation for 2 h at 4 °C in a rotating wheel, gamma-bind Sepharose-protein G was added (Amersham Biosciences), followed by incubation for 1 h at 4 °C. The complexes were harvested by centrifugation and washed three washes with RIPA buffer, resolved in SDS–polyacrylamide gels, and immunobloted. Mouse IgG TrueBlot (eBiosciences) was used as a peroxidase-conjugated secondary antibody (1:10,000 dilution) because it reduces interference by the 55-kDa heavy and 23-kDa light chains of the immunoprecipitation antibody.

#### Lambda Phosphatase Assay

HEK293T cells were co-transfected with Nurr1^WT^-V5/6xHis and pCGN-HA-GSK-3β^Δ9^ or pCGN-HA-GSK-3β^Y216F^ using TransFectin Lipid Reagent (Bio-Rad) according to manufacturer recommendations. After 24 h of recovery from transfection, the cells were lysed in 200 μl lysis buffer (137 mM NaCl, 20 mM Tris–HCl, pH 7.5, 1% Nonidet P40, 10% glycerol, 1 μg/ml leupeptin, 1 mM phenylmethylsulfonyl fluoride). Then, the samples were sonicated and precleared by centrifugation, and 50 μl of the sample was incubated with λ-protein phosphatase (Upstate, Millipore) for 4 h at 37 °C. Then, the samples were resolved by SDS-PAGE and immunoblotted.

#### Two-Dimensional Electrophoresis

HEK293T cell co-transfected with expression plasmids for Nurr1^WT^-V5/6xHis, Nurr1^MUT2^-V5/6xHis and pCGN-HA-GSK-3β^Δ9^ or pCGN-HA-GSK-3β^Y216F^, using TransFectin Lipid Reagent (Bio-RAD) according to manufacturer recommendations. For experimental details, see Supplemental Material.

#### Analysis of mRNA Levels

Total RNA was extracted using TRIzol reagent according to the manufacturer’s instructions (Invitrogen). Reverse transcription and quantitative PCR were done as detailed elsewhere [44]. Primer sequences are shown in Supplemental Table 3. Data analysis was based on the ΔΔCT method with normalization of the raw data to housekeeping genes *Actb* and *Gapdh* (Applied Biosystems, Thermo Fisher Scientific). All PCRs were performed in triplicate.

#### Luciferase Assays

Luciferase activities were determined using a luciferase assay system (Promega) as per the manufacturer’s instructions. As a reference plasmid to normalize transfection efficiency, a CMV-galactosidase plasmid (Promega) was cotransfected in all experiments and luciferase assay values were normalized to galactosidase activity.

#### Statistics

Results are expressed as mean ± SEM from at least three independent experiments. Data were analyzed by one-way ANOVA followed by Newman–Keuls multiple comparison test (*p* ≤ 0.001), or with Student’s *t* test (*p* ≤ 0.05), using Prism version 5.03 software (GraphPad, San Diego, CA, USA).

## Results

### α-SYN Aggregates Reduce the DAergic Phenotype of SH-SY5Y Cells

**Fig. 1:**
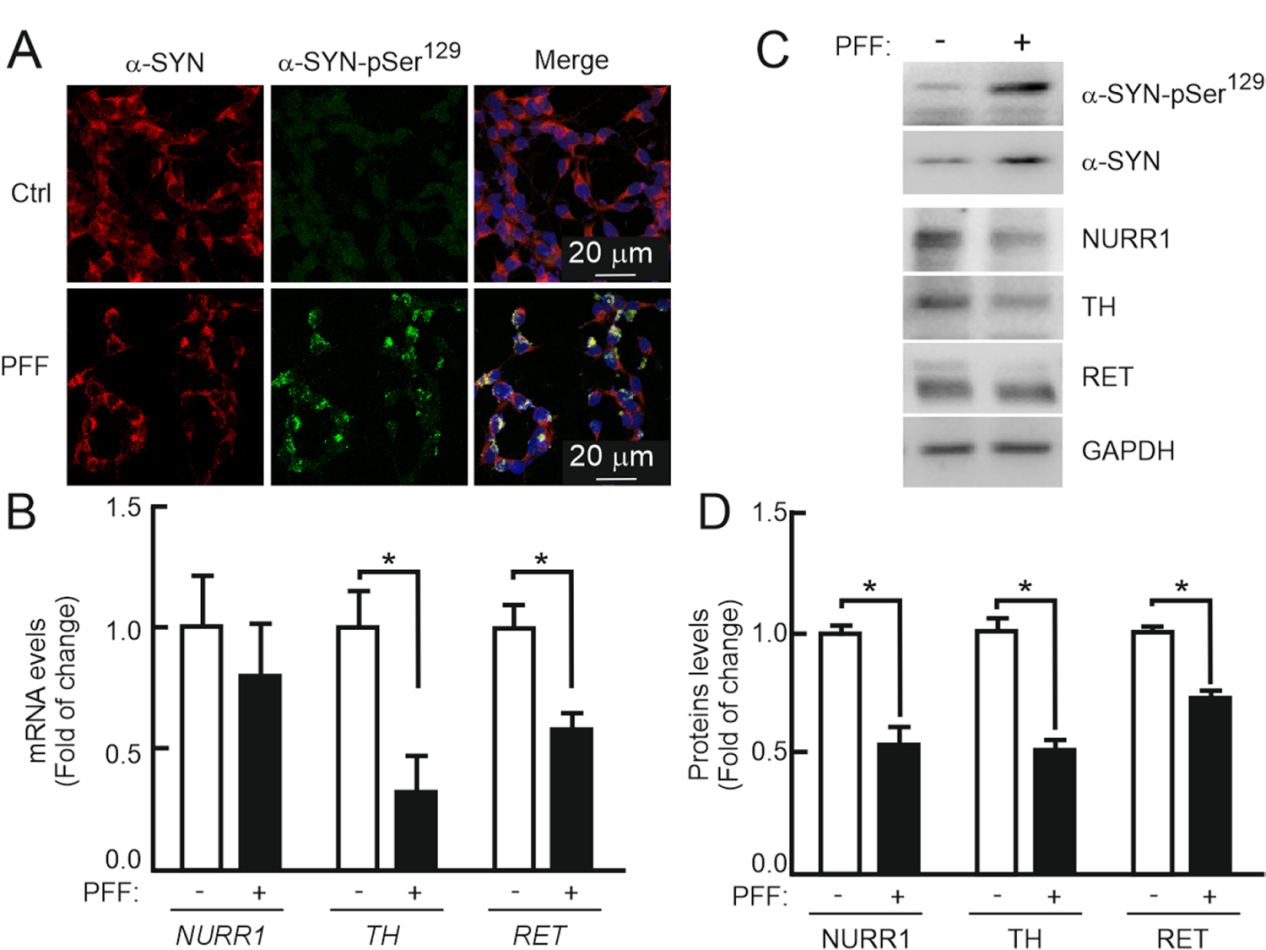
Pre-formed fibrils (PFF) of α-SYN disturb the DAergic phenotype. **A** Confocal immunofluorescence of SH-SY5Y cells submitted to PFF (1 µg/ml PFF, 10 days). Note the presence of cytoplasmic α-SYN-pSer^129^ aggregates in the presence of PFFs. **B** qRT-PCR determination of transcript levels for *NURR1*, *TH*, and *RET* normalized by the average of housekeeping genes *ACTB* and *GAPDH*. Values are mean ± S.E.M. (*n* = 3). Statistical analysis was performed with Student’s *t* test. *p < 0.05 vs. untreated cells. **C** Immunoblots of cells treated under the same conditions. Upper two panels, anti-α-SYN-p-Ser^129^ and anti-total α-SYN antibodies. Lower four blots, anti-NURR1, anti-TH, anti-RET, and-GADPH antibody used as a protein loading control. **D** Densitometric quantification of NURR1, TH, and RET protein levels relative to GAPDH is representative blots of C. Data are mean ± SEM (*n* = 3). Statistical analysis was performed with Student’s t test. *p < 0.05 vs. untreated cells

In order to identify the mechanism involved in the dysregulation of the DAergic phenotype, we incubated the DAergic cell line SH-SY5Y with preformed fibrils (PFFs) of human recombinant α-SYN (1 µg/ml, 10 days). Confocal microscopy demonstrated the formation of aggregates containing α-SYN and Ser^129^-phopshorylated α-SYN (α-SYN-pSer^129^) (Fig. 1A). PFFs induced a slight nonsignificant decrease in *NURR1* transcript levels compared to the control untreated cells, indicating similar *NURR1* gene expression, but at the same time, *TH* and *RET* transcripts were diminished (Fig. 1B). By contrast, not only TH and RET proteins were decreased but also NURR1 (Fig. 1C and D). The fact that *NURR1* gene expression was little or no affected by PFFs (see “Discussion”), together with the decrease in NURR1 protein levels, suggests that α-SYN PFFs must cause, at least in part, a reduction of NURR1 protein stability and subsequent decrease in the expression of NURR1 target genes, such as *TH* and *RET*.

**Fig. 2:**
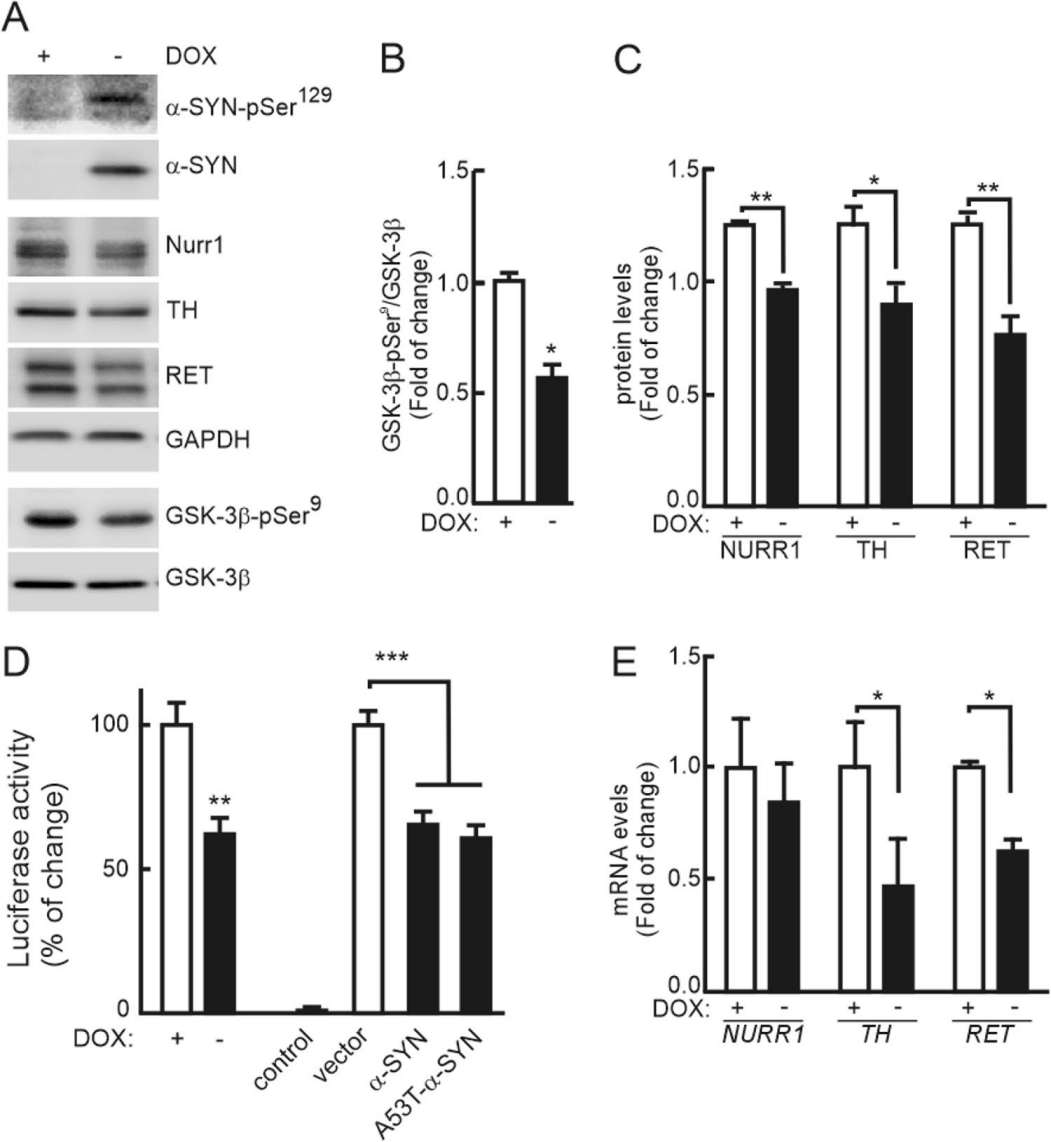
Inducible expression of α-SYN impairs the DAergic phenotype. **A** Immunoblots of SH-SY5Y cells, expressing α-SYN under the control of the Tet-Off system. Cells were treated with vehicle or DOX (2 µg/ml, 5 days) in the presence of RPMI with serum. Then, cells were transferred to Opti-MEM Reduced Serum maintaining the DOX treatment for 8 days. **B**, **C** Densitometric quantification of GSK-3-pSer^9^ normalized by total GSK-3 and NURR1, TH, and RET protein levels normalized by GADPH, respectively. Values are the mean ± SEM (*n* = 3). Statistical analysis was performed with a Student’s *t* test. *p < 0.05, **p > 0.01 vs. DOX-treated groups. **D** Leftmost bars, transcriptional activity of endogenous NURR1 measured by luciferase assay after transient transfection of an NBRE-luciferase reporter construct (with and without α-SYN overexpression). Rightmost bars, myc-NURR1 transcriptional activity after co-transfection of NBRE-luciferase, and either CMV-driven wild-type α-SYN or A53T-α-SYN. **E** Tet-Off SH-SY-5Y-α-SYN cells were transfected with a CMV-driven myc-NURR1 expression. The graph shows qRT-PCR determination of transcript levels of *NURR1*, its targets *TH* and *RET* normalized by the average of housekeeping genes *ACTB* and *GAPDH*. Values are mean ± S.E.M. (*n* = 3). Statistical analysis was performed with Student’s *t* test. *p < 0.05

We further analyzed the regulation of NURR1 in the Tet-Off SH-SY5Y cell line, conditionally expressing α-SYN in the absence of doxycycline (DOX) [46]. DOX removal from the culture medium led to a robust expression of α-SYN after 8 days, and α-SYN and α-SYN-pSer^129^ expression correlated with a decrease in NURR1, TH, and RET protein levels (Fig. 2A-C). Moreover, α-SYN overexpression resulted in a reduction of the NURR1 target genes *TH* and *RET* (Fig. 2B). To further determine the effect on gene expression, first we transfected these cells with a NBRE-luciferase reporter-construct, specifically activated by NURR1. Upon DOX removal, α-SYN overexpression correlated with reduced luciferase levels (Fig. 2D, left bars), suggesting that NURR1 activity parallels NURR1 protein levels. Similar results were obtained when naïve SH-SY5Y cells were co-transfected with the NBRE-luciferase reporter and expression vectors for wild type or A53T-α-SYN (Fig. 2D, right bars). This effect was not observed upon transfection of the control vector. We further expressed in the Tet-off SH-SY5Y cells, a myc-tagged *NURR1* cDNA under the control of a heterologous CMV promoter (Fig. 2E). *NURR1* transcript levels arising from the construct were unaffected by α-SYN overexpression, yet *TH* and *RET* expression were decreased. These experiments further verify that α-SYN reduces NURR1 protein levels and the expression of its target genes.

### GSK-3 Is Needed to Downregulate NURR1

**Fig. 3:**
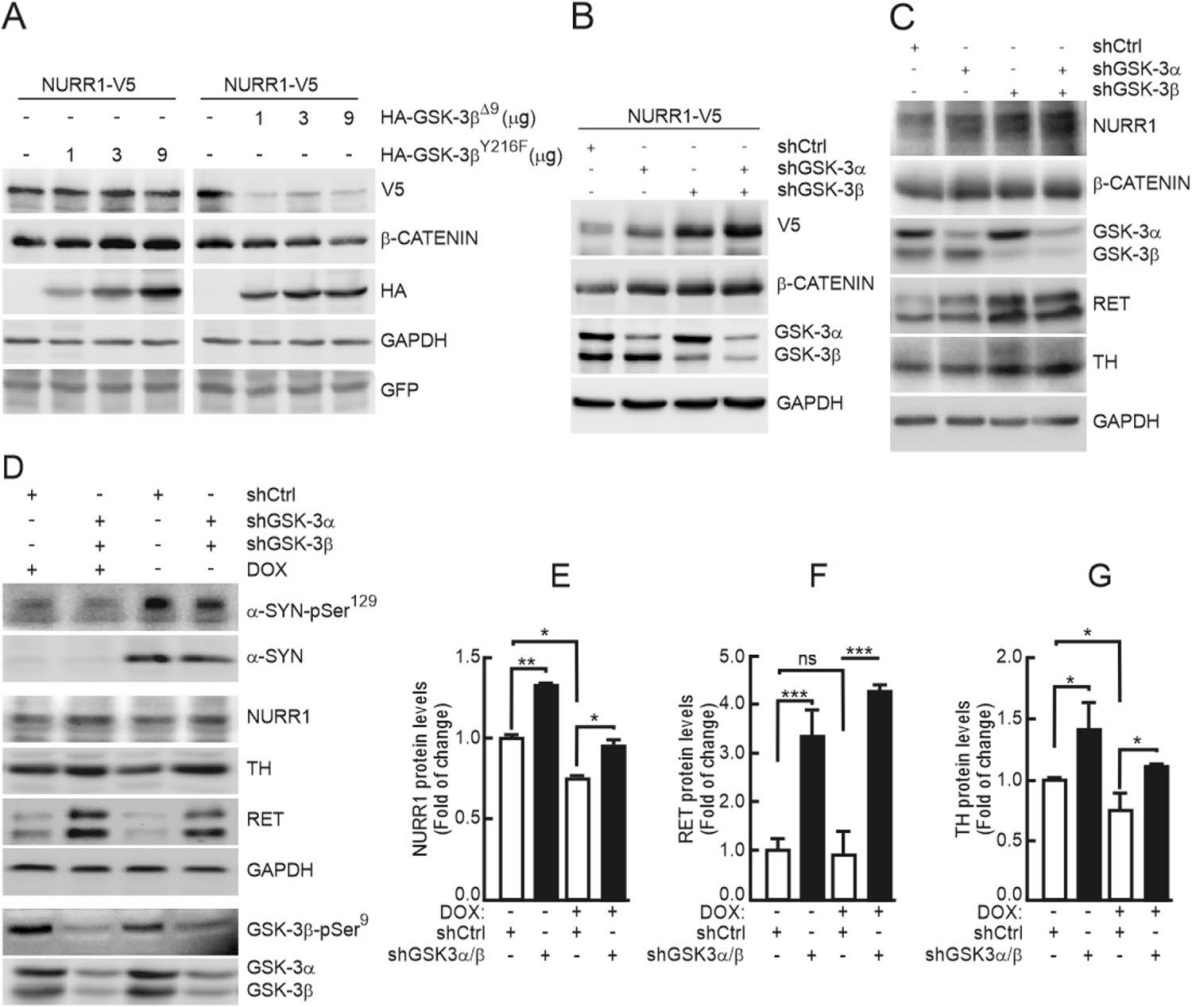
GSK-3 is instrumental in α-SYN-induced NURR1 downregulation. **A** Reduction of NURR1 levels in the presence of of GSK-3β. HEK293T cells were co-transfected with the NURR1^WT^-V5 expression vector and the indicated amounts of the HA-GSK-3β^Y216F^ mutant and dominant-positive HA-GSK-3β^Δ9^ mutant, and then maintained in low-serum medium for 16 h. GFP expression was used as control for even transfection. Whole-cell lysates were immunoblotted against anti-V5 antibody (NURR1) or anti-HA antibody (GSK-3β). β-Catenin levels were immunoblotted as a control for GSK-3 activity and anti-GAPDH antibody as control of protein load. **B** HEK293T cells were infected with an expression vector for V5-tagged NURR1 and the lentiviruses specific shCtrl, shGSK-3α, and shGSK-3β. Upper blots, V5-NURR1 and β-catenin protein levels. Lower blots, GSK-3α/β and GAPDH protein levels. **C** Naïve SH-SY5Y cells were infected with lentiviruses that carried a specific silencer for a control scramble sequence (shCtrl), GSK-3α (shGSK-3α) or GSK-3β (shGSK-3β). After 24 h, cells were transferred to Opti-MEM Reduced Serum Medium, and grown for 3 days. Upper blot, NURR1 protein levels. β-Catenin levels were used as positive control of GSK-3 activity. In the middle blots, GSK-3α/β protein levels as control shRNA treatment, and TH and RET protein levels as NURR1 targets. Lower blot, GAPDH protein levels showing similar protein load per lane. **D** Tet-Off SH-SY5Y cells were treated with vehicle or DOX (2 µg/ml, 5 days) in RPMI with serum. Then, the cells were infected with shCtrl, shGSK-3α and shGSK-3β lentiviruses. After 24 h, cells were transferred to Opti-MEM Reduced Serum Medium for 8 days maintaining the DOX treatment. Upper panels, tetracycline-induced α-SYN and its α-SYNpSer^129^. Middle panels, expression of endogenous NURR1, TH and RET and GAPDH. Lower panels, levels of GSK-3β-pSer^9^ and total levels of GSK-3α/β. **E**, **F**, **G** Quantitative determination of NURR1, RET, and TH levels, respectively, normalized by GADPH levels. Values represent mean ± SEM (*n* = 3). A Student’s *t* test was used to assess difference among groups. *p < 0.05, **p < 0.01, ***p < 0.001, ns, non-significant

α-SYN overexpression reduced the levels of GSK-3β phosphorylated at Ser^9^ (GSK-3β-pSer^9^) (Fig. 2A, B). This phosphoserine exerts an inhibitory effect on the kinase by blocking the catalytic site [47, 48]. Therefore, reduction in pSer^9^-GSK-3 levels is indicative of increased kinase activity. To determine if increased GSK-3 activity might affect NURR1 stability, we first used the easy-to-transfect human cell line HEK293T. Transcriptomic data indicate that these cells originated from the neural crest and express several neuron-specific genes [49, 50]. We ectopically expressed a V5-targed NURR1 together with constitutively active GSK-3β lacking the first nine amino-terminal residues that correspond to the pseudosubstate (GSK-3β^Δ9^). Lack of Ser^9^ renders this kinase insensitive to downregulation by AKT-mediated phosphorylation. As a negative control, we used a hypomorphic mutant containing a single point Tyr-to-Phe mutation in its activation loop (GSK-3β^Y216F^) that renders this kinase almost inactive [51, 52]. As shown in (Fig. 3A), NURR1 levels were reduced with increasing amounts of the GSK-3β^Δ9^, and were not altered in the presence of inactive GSK-3β^Y216F^. Then, we silenced the expression of both α and β isoforms of this kinase by lentiviral knock-down for 3 days in V5-NURR1 expressing HEK293T cells (Fig. 3B) as well as in naïve SH-SY5Y cells (Fig. 3C). As a control, we analyzed β-catenin levels, a well-established substrate of GSK-3, which is degraded upon GSK-3β-mediated phosphorylation. Just a ~ 50% decrease in GSK-3α and GSK-3β levels increased NURR1 levels and its targets RET and TH. We also silenced both isoforms in the Tet-Off SH-SY5Y-α-SYN cells (Fig. 3D-G). Despite α-SYN overexpression, GSK-3α and GSK-3β knock-down rescued NURR1 levels to baseline as well as TH and RET levels. The effect was most evident in the GSK-3β knocked-down cells, indicating a preponderant role for this isoform. These results show for the first time that GSK-3 is required for the downregulation of NURR1 induced by α-SYN and are in line with observations based on GSK-3 inhibitors in the Parkinsonian MPTP and MPP^+^ [53, 54], or 6-OHDA [55–57] models. The mechanisms of action of these two toxins, altering mitochondrial activity and redox homeostasis, is at least partially different to the proteinopathy elicited by α-SYN aggregates, therefore suggesting that these different pathomechanisms overlap on the GSK-3/NURR1 axis reported here for α-SYN.

### GSK-3 Targets NURR1 for Degradation

**Fig. 4:**
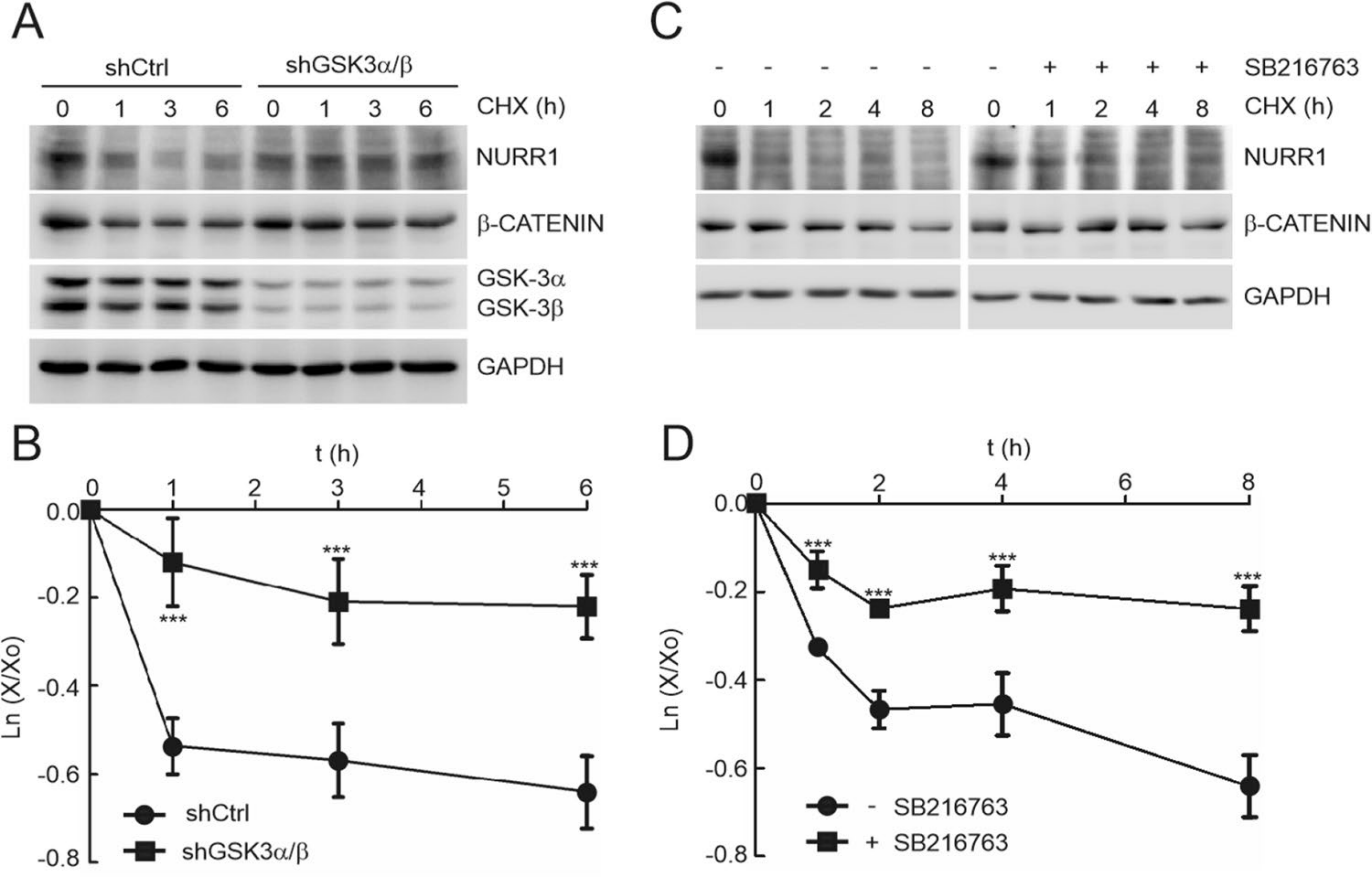
GSK-3 reduces the half-life of NURR1. **A** SH-SY5Y were infected with lentiviral silencers shCtrl, shGSK-3α, and shGSK-3β in RPMI with serum. After 24 h, cells were transferred to Opti-MEM Reduced Serum Medium for 3 days. Finally, the cells were treated with 100 µM CHX for the indicated times. Upper blots, NURR1 protein levels, and β-catenin protein levels used as positive control GSK-3 activity. Middle blots, GSK-3α/β protein levels, as control shRNA knock-down. GAPDH protein levels, as protein loading control. **B** Graph depicts the natural logarithm of the relative levels of the NURR1 protein as a function of CHX chase time in SH-SY5Y cells treated like in **A**. **C** Cells were maintained in Opti-MEM Reduced Serum Medium for 16 h and then treated with the GSK-3 inhibitor SB216763 (5 μM, 2 h) prior to inhibition of protein synthesis with CHX. **D** Graph depicts the natural logarithm of the relative levels of the NURR1 protein as a function of CHX chase time in SH-SY5Y cells treated like in **C**. For **B** and **D**, the protein half-life was determined in the linear range of the degradation curve. Statistical analysis was performed with one-way ANOVA followed by Newman–Keuls multiple comparison test. ***p < 0.001

We next examined the effect of GSK-3 on NURR1 turn-over. SH-SY5Y cells were infected with lentiviral vectors expressing shCTRL, shGSK-3α/shGSK-3β. After 3 days, cells were incubated with the protein synthesis inhibitor cycloheximide (CHX, 100 µM) (Fig. 4A, B). As a control, we monitored the stability of β-catenin. In shCTRL cells, NURR1 had a half-life of ~ 6 h. However, in the GSK-3-knocked-down cells NURR1 was almost completely stable during this time. As an additional approach, we performed similar experiments but inhibiting GSK-3 with the potent and selective inhibitor SB216763 (5 µM, pre-incubated for 2 h) (Fig. 4C, D). In vehicle-treated cells, NURR1 exhibited a half-life of ~ 6 h, as observed before, but in SB216763-treated cells, the levels of NURR1 were hardly affected. Therefore, both genetic and chemical inhibition of GSK-3 results in stabilization of NURR1.

### GSK-3β Targets NURR1 for Phosphorylation and Degradation Through the UPS

**Fig. 5:**
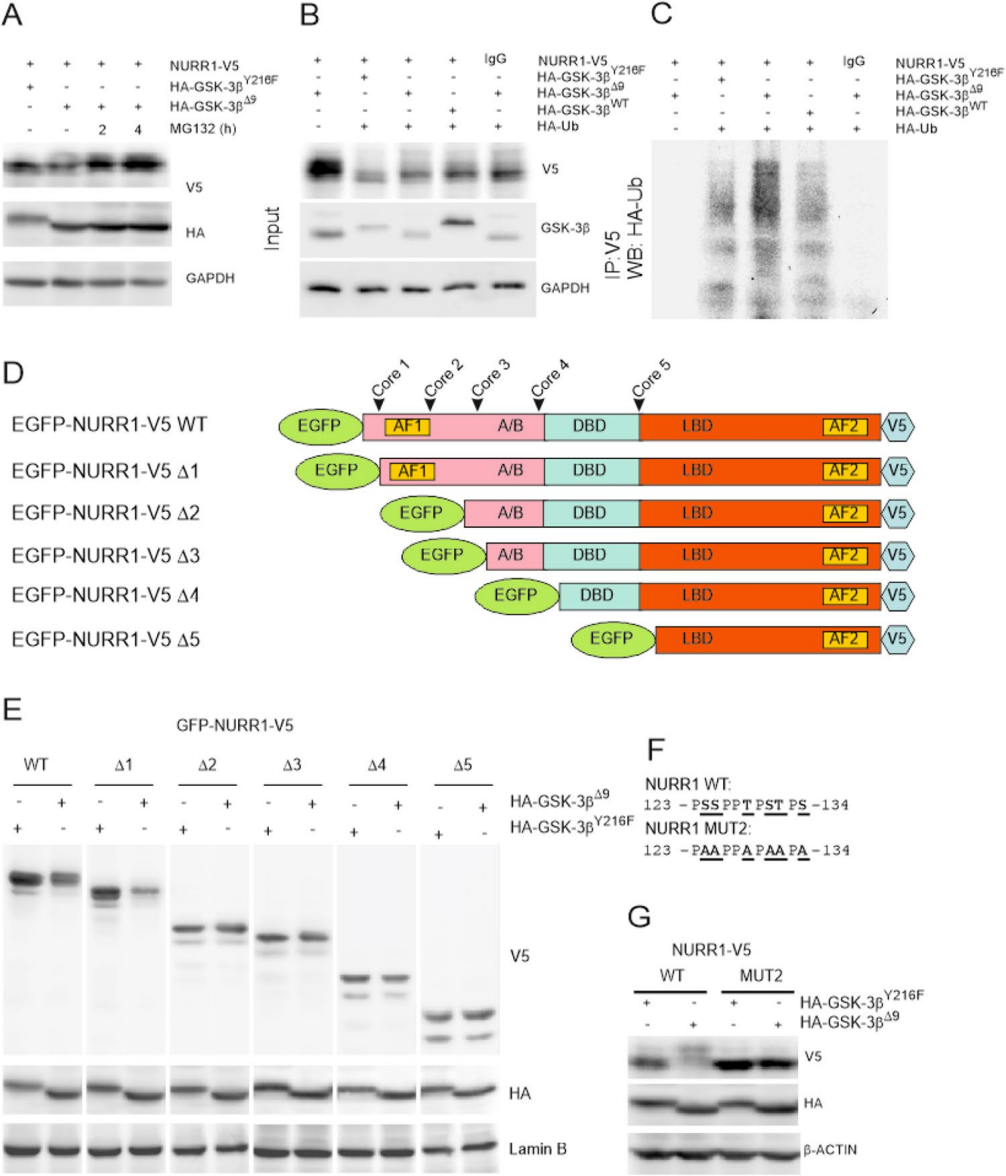
GSK-3 induces NURR1 phosphorylation and degradation. **A** HEK293T cells were co-transfected with expression vectors for NURR1-V5 and hypomorphic HA-GSK-3β^Y216F^ or active HA-GSK-3β^Δ9^. After 16 h, cells were subjected to the ubiquitin–proteasome inhibitor MG132 (20 µM, for 2 h and 4 h). Upper blot, anti-V5 antibody showing ectopically expressed NURR1-V5; middle panel, anti-HA antibody showing the GSK-3β proteins. Note the smaller size of HA-GSK-3β^Δ9^ due to the deletion of the first nine N-terminal residues. Lower blot, GAPDH levels showing similar protein load per lane. **B**, **C** Ubiquitination assay. HEK293T cells were co-transfected with the indicated plasmids and HA-tagged ubiquitin (HA-Ub) expression vector. One fifth of whole-protein lysate was used as input to control for protein expression (**B**). The rest of the protein lysates were immunoprecipitated with anti-V5 antibody and immunoblotted with anti-HA (Ub) indicated in **C**. **D** Schematic illustration of NURR1-V5 chimeras used for mapping GSK-3-sensitive sites in NURR1. EGFP, enhance green fluorescent protein; A/B (AF1), DBD, LBD (AF2), V5, C-terminal tag used for detection in immunoblot. **E** HEK293T cells were co-transfected with EGFP-NURR1^WT^-V5 and the chimeric deletion mutants, along with HA-GSK-3β^Y216F^ or HA-GSK-3β^Δ9^ expression vectors, and then maintained in low-serum medium for 16 h. Whole-cell lysates were immunoblotted against anti-V5 antibody (EGFP-NURR1 chimeras) or anti-HA antibody (GSK-3β). Lamin B levels show similar protein load per lane. **F** Amino acid sequence of Core2 in wild-type NURR1 and the alanine substitutions mutated to generate NURR1^MUT2^-V5. **G** HEK293T cells were co-transfected with NURR1^WT^-V5 and NURR1^MUT2^-V5, along with HA-GSK-3β^Y216F^ or HA-GSK-3β^Δ9^ as indicated. Then, cells were maintained in low-serum medium for 16 h before immunoblotting with the indicated antibodies

HEK293T cells were co-transfected with expression vectors for NURR1-V5 and HA-GSK-3β^Δ9^, or HA-GSK-3β^Y216F^ as control. After 16 h, the cells were treated for 2 h and 4 h with the selective proteasome inhibitor MG132 (20 µM). As shown in Fig. 5A, UPS inhibition protected NURR1 from GSK-3β-mediated degradation. In an ubiquitination assay, HEK293T cells were co-transfected with expression vectors for NURR1-V5 along with HA-tagged ubiquitin and either HA-GSK-3β^Y216F^, or HA-GSK-3β^WT^ or HA-GSK-3β^Δ9^ (Fig. 5B, C). Overexpression of GSK-3β^WT^ slightly ubiquitylated NURR1, and constitutively active GSK-3β^Δ9^ considerably enhanced ubiquitination. It has been reported previously that NURR1 is degraded by the UPS [22, 58, 59], but our results show for the first time the direct participation of GSK-3β.

GSK-3 phosphorylates its substrates in two specific consensus sequences, (Ser/Thr)-Pro or (Ser/Thr)-X_3_-(pSer/pThr), where X is any residue [60]. Using the NetPhos 2.0 program, we found that NURR1 contains at least five putative sequences with serines or threonines that conform to the consensus motif for GSK-3 phosphorylation. We named these sites, Core 1, 2, 3, 4, and 5 (Fig. 5D and Supplemental Fig. 1A). Then, we generated sequential deletion mutants fused to enhanced green fluorescence protein (EGFP) at the N-terminus and a V5 tag at the C-terminus (Fig. 5D) and analyzed their phosphorylation pattern (Fig. 5E). GSK-3β^Δ9^ induced a slightly retarded EGFP-NURR1 band in the full length chimera and in the first deletion mutant (Δ1), compared to control GSK-3β^Y216F^. In addition, the protein levels of these two constructs were decreased in the presence of GSK-3β^Δ9^. By contrast, the rest of the EGFP-NURR1 mutants exhibited low or no obvious band shift and were resistant to degradation in the presence of GSK-3β^Δ9^, suggesting that the sites targeted by GSK-3β on NURR1 are preferentially located before or at Core 2 (Fig. 5E). As control, EGFP alone was insensitive to GSK-3β-induced band shift or degradation (data not shown). Additionally, we performed point mutations of NURR1 at Core 2 by changing 4 serines and 2 threonines to alanines (Fig. 5F). We found that Core 2 mutation rendered NURR1 insensitive to GSK-3β-induced band shift and degradation (Fig. 5G). The amino acid sequence of Core 2, comprising residues 123 to 134 is highly conserved in vertebrates (Supplemental Fig. 1B-C).

**Fig. 6:**
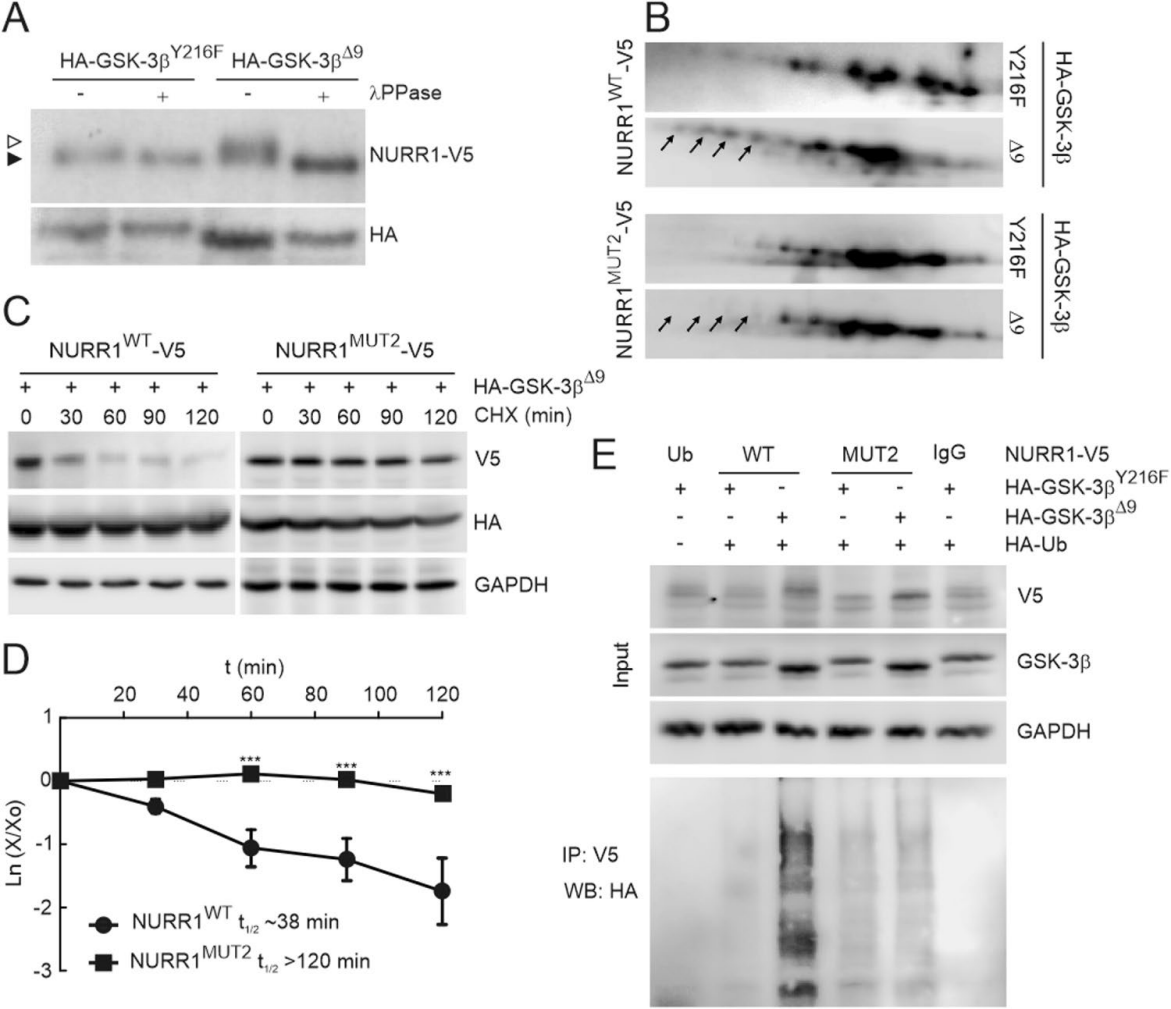
GSK-3 induces the phosphorylation of NURR1. **A** Lambda phosphatase assay. Cells were co-transfected with expression vector for NURR1-V5 and either HA-GSK-3β^Y216F^ or HA-GSK-3β^Δ9^. Cell lysates were incubated with or without λ-phosphatase as indicated. Empty arrowhead, retarded band that corresponds to phosphorylated NURR1; black arrowhead, non-phosphorylated NURR1. **B** Analysis of NURR1 phosphorylation by 2D-PAGE. HEK293T cells were co-transfected with NURR1^WT^-V5 and NURR1^MUT2^-V5, along with HA-GSK-3β^Y216F^ or HA-GSK-3β^Δ9^, and then maintained in low-serum medium for 16 h. 2D-PAGE immunoblots were revealed against anti-V5 antibody (NURR1). Black arrows indicate acidic spots that result from GSK-3 phosphorylation and are lost in NURR1^MUT2^-V5. **C**, half-life of NURR1^MUT2^-V5 is not affected by GSK-3β. HEK293T cells were co-transfected with NURR1-V5, NURR1^MUT2^-V5 together with HA-GSK-3β^Y216F^ or HA-GSK-3β^Δ9^, serum starved for 16 h, and finally incubated for the indicated time with 100 µM CHX. Upper blot, anti-V5 (NURR1^WT^-V5 or NURR1^MUT2^-V5) protein levels. Middle blot, HA-GSK-3β mutants. Lower blot, GAPDH levels showing similar protein loads per lane. **D**, the graph depicts the natural logarithm of the relative levels of the NURR1^WT^-V5 and NURR1^MUT2^-V5 protein as a function of CHX incubation time. The protein half-life was determined using the linear part of the degradation curve. Statistical analysis was performed with one-way ANOVA followed by Newman–Keuls multiple comparison test. ***p < 0.001. **E** Ubiquitilation of NURR1^MUT2^-V5 is drastically reduced despite the presence of active GSK-3β. HEK293T cells were co-transfected with the indicated plasmids or without HA-Ub vector (Ub) as control. One fifth of whole-protein lysate was used to control for protein expression as shown in the three upper panels (total Input). The rest of the protein lysates were immunoprecipitated with anti-V5 antibody and immunoblotted as anti-HA to detect ubiquitinated NURR1

To confirm that GSK-3β induces NURR1 phosphorylation, we performed a lambda-phosphatase assay (λPPase) in HEK293T cells co-transfected with expression vectors for NURR1-V5 and GSK-3β^Δ9^ or GSK-3β^Y216F^ (Fig. 6A). In the presence of GSK-3β^Δ9^, NURR1 showed a retarded band in SDS-PAGE. This gel shift was abrogated when the protein lysate was incubated with the phosphatase, therefore demonstrating that the retarded band is due to GSK-3-mediated phosphorylation.

To more precisely characterize the relevance of Core 2, HEK293T cells were transfected with NURR1^WT^ or NURR1^MUT2^ and co-transfected with GSK-3β^Δ9^ or GSK-3β^Y216F^, and resolved by 2D gel electrophoresis. As shown in Fig. 6B, NURR1^WT^ co-transfected with hypomorphic GSK-3β^Y216F^, displayed several immunoreactive spots consistent with GSK-3β-independent posttranslational modifications of NURR1. When cells were co-transfected with active GSK-3β^Δ9^, we observed an increase in the intensity of acidic spots (black arrows), indicating GSK-3-mediated phosphorylation. However, in cells co-transfected with NURR1^MUT2^ the distribution of spots was similar in the presence of GSK-3β^Y216F^ or GSK-3β^Δ9^, and the acidic shift was not observed (black arrows). These results indicate that the residues of Core 2 are preferred targets of GSK-3β phosphorylation.

In additional experiments, we examined the half-life of NURR1^MUT2^. HEK293T cells were co-transfected with NURR1^WT^ or NURR1^MUT2^ and GSK-3β^Δ9^, and exposed to CHX (100 µM) (Fig. 6C, D). In the presence of GSK-3β^Δ9^, the half-life of NURR1^WT^ was less than 120 min. However, NURR1^MUT2^ exhibited a half-life of more than 120 min that was not significantly shortened by the presence of GSK-3β^Δ9^. We also performed an ubiquitination assay in HEK293T cells co-transfected with expression vectors for NURR1^WT^ or NURR1^MUT2^ along with HA-Ubiquitin and GSK-3β^Y216F^ or GSK-3β^Δ9^. As shown in Fig. 6E, overexpression of GSK-3β^Δ9^ enhanced NURR1^WT^ ubiquitination, while in NURR1^MUT2^ this was less apparent.

**Fig. 7:**
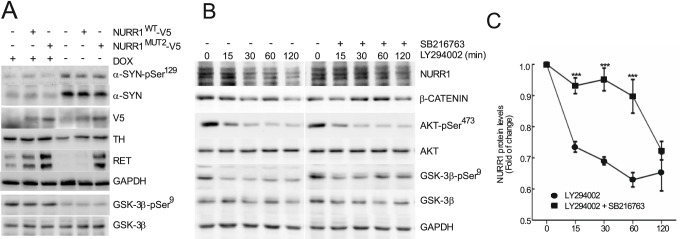
GSK-3β activation by conditional expression of α-SYN or by phosphatidylinositol-3 kinase (PI3K) inhibition leads to loss of NURR1 stability. **A** SH-SY5Y cells carrying the α-SYN Tet-Off expression system were treated with or without 2 µg/ml DOX for 5 days in the presence RPMI with serum. Then, the cells were infected with lentiviruses expressing to NURR1^WT^-V5 and NURR1^MUT2^-V5. After 24 h, cells were transferred to Opti-MEM Reduced Serum Medium maintaining the DOX treatment for 8 days. **B** SH-SY5Y cells were maintained in Opti-MEM Reduced Serum Media for 16 h and then pre-treated with SB216763 (5 μM, 2 h) prior to inhibition of the PI3K/AKT pathway with 30 µM LY294002. β-Catenin protein levels were used as positive control GSK-3 activity. AKT-pSer^473^ and GSK-3β-pSer^9^ levels were used as control LY294002 treatment. Finally, GAPDH protein levels show similar protein loaded per lane. **C** Densitometry quantification of NURR1 protein levels normalized with GAPDH from representative blots of cells treated like in **B**. Each value is the mean ± S.E.M. (*n* = 3). Statistical analysis was performed with one-way ANOVA followed by Newman–Keuls multiple comparison test. ***p < 0.001

Finally, we validated the phosphorylation of Core 2 in the DAergic cell line SH-SY5Y (Fig. 7A). These cells were infected with lentiviral vectors expressing NURR1^WT^ or NURR1^MUT2^. In the presence of DOX, α-SYN levels were low in cells expressing either form of NURR1, and the levels of GSK-3β-pSer^9^ were high, indicating its inhibition. However, in the absence of DOX, α-SYN levels increased, and GSK-3 was dephosphorylated and active. The presence of α-SYN and its downstream target, active GSK-3, completely eliminated NURR1^WT^ protein and substantially reduced the levels of TH and RET. By contrast, the levels of NURR1^MUT2^ were not affected by the α-SYN/GSK-3 challenge and the levels of TH and RET remained similar to those in the absence of α-SYN.

A highly characterized survival pathway in nerve cells is the PI3K/AKT. This pathway is activated by many growth factors and neurotrophins and leads to the inhibition of GSK-3. Therefore, we analyzed the inhibition of this pathway, by using the highly selective PI3K inhibitor LY294002. SH-SY5Y cells were submitted to a time-course of LY294002 (30 µM) alone or in combination with the GSK-3 inhibitor SB216763 (5 μM). As shown in Fig. 7B, C, LY294002 alone led to a decrease in AKT-pSer^473^ (inactivation) and GSK-3β-pSer^9^ (activation). Under these conditions, not only β-catenin but also NURR1 levels were gradually decreased. By contrast, cells co-treated with SB216763 were at least partially protected from the decrease in β-catenin and NURR1. Together, these observations confirm in a DAergic cell line the mechanistic connections between α-SYN, GSK-3β, phosphorylation, and degradation of NURR1.

## Discussion

The dysfunction of the DAergic nigrostriatal tract in PD proceeds in two phases: first, loss of the DAergic function of neurons of the SN pars compacta and then death of these neurons. The two phases can be separated by exposure to toxins that induce a transient loss of the DAergic phenotype including amphetamine, MPTP, and α-SYN [17, 61–65]. The protection and functional recovery of these neurons is essential to endorse an early neuroprotective therapy, but such a strategy needs a fine knowledge of the molecules that participate in maintaining the DAergic phenotype during adulthood and the mechanisms that subvert their activity [66, 67].

Here, we focused our study on NURR1 because α-SYN inhibits expression of DAergic genes probably by altering NURR1 expression or activity [68–70]. Those studies and also ours assessed the role of aggregated α-SYN, but we cannot discard at this time that monomeric α-SYN might also participate in GSK-3 activation. In fact, ectopic overexpression of α-SYN elicited a reduction in *NURR1* expression as indicated by the luciferase reporter (Fig. 2D) that might be related to monomeric α-SYN, although we cannot determine if a fraction of exogenously expressed α-SYN was aggregated. Furthermore, in the Tet-off inducible model in SH-SY5Y cells used here [46], α-SYN produces Triton-soluble and insoluble oligomers, which could be responsible for regulation of the GSK-3/NURR1 axis. Further studies are required under very carefully controlled conditions to finely determine the contribution of monomeric, oligomeric soluble and insoluble, and fibrillary forms of α-SYN to the regulation of GSK-3 and NURR1 protein levels.

Thus, two recent postmortem studies show that the levels RET receptor are reduced by about 80% in nigral neurons containing α-SYN inclusions, leading to impaired RET signaling [71] and probably explaining the limited benefit of GDNF/NRTN-therapeutics in humans [71] and rodent models [72]. This reduction is most likely linked to impaired NURR1 activity because conditional expression of mutant α-SYN in the midbrain DA neurons causes NURR1 degradation and progressive neurodegeneration [22].

α-SYN might inhibit *NURR1* gene expression, promote NURR1 protein degradation, or both. The NURR1 coding gene contains an NF-kB site in its promoter, and it has been reported that α-SYN down-regulates NURR1 through inhibition of NF-κB [73]. However, in our cellular remodels, we have detected a very minor reduction in NURR1 mRNA levels. Our results are in line with other studies that report exacerbated NURR1 degradation by the UPS, and NURR1 stabilization via proteasomal inhibition ameliorates degeneration of mDA neurons induced by α-synuclein [22, 74]. Considering that α-SYN is expressed in many tissues and cells, it is likely that different forms of NURR1 regulation operate under specific conditions.

Previous reports have found that GSK-3 phosphorylates α-SYN in a similar manner to TAU [33, 36]. More relevant to our study, α-SYN appears to activate GSK-3β by ill-defined mechanisms that might be related to the formation a complex containing α-SYN/GSK-3β/TAU [27] or by downregulation of signaling pathways such as retinoic acid [24]. Thus, GSK-3β is robustly activated in MPTP models of Parkinsonism, in transgenic mice overexpressing α-SYN, and in the striatum of PD patients [28]. However, we show for the first time that activation of GSK-3β by aggregated α-SYN leads to phosphorylation and subsequent UPS degradation of NURR1, leading to loss of DAergic markers. Mechanistically, we identify a region between 123 and 134 amino acids, harboring proline-directed residues, as the main target of this kinase. Thus, we identify Core 2 as a phosphorylation-dependent degradation domain, phosphodegron, which leads to its UPS degradation.

Human NURR1 contains 61 serines, 27 threonines, and 20 tyrosines distributed along 598 amino acids, and most likely, it is submitted to posttranslational modifications by several protein kinases. NURR1 phosphorylation has been reported to occur by AKT at Ser^347^ leading to increased protein stability [59], and by RSK/MSK at the same residue [75]. Nine of the putative phosphorylation residues are followed by a proline, and might be phosphorylated by proline-directed kinases such as MAP kinases. In fact, ERK2 phosphorylates NURR1 on multiple sites in in vitro kinase assays, including Ser^126^ and Thr^132^, which are located at Core 2. Luciferase reporter assays with a reporter plasmid containing 1 kb of the TH promoter further suggested that these phosphosites are required for ERK2 regulation in SH-SY5Y cells [76]. Contrary to ERK2, GSK-3 is a kinase that remains inactive in the presence of serum and requires growth factor deprivation such as GDNF-deficiency for its activation [77]. Therefore, it is conceivable that Core 2 functions as a molecular switch in coordination with other signals that ultimately will lead to NURR1 activation or to proteasomal degradation. On the other hand, many GSK-3 substrates need to be previously phosphorylated by another kinase in order to be recognized by GSK-3. Therefore, it is also possible that an initial phosphorylation, originated by ERK, might lead to transient NURR1 activation and, at the same time, the ERK-phosphorylated NURR1 would be primed for degradation at a later stage when cell signalling is reduced and GSK-3 becomes active. Such a mechanism would provide a control for over-activation of NURR1. However, under pathological conditions, where neurotrophin signaling is limiting, an imbalance in GSK-3 activity would favor NURR1 degradation over activation.

Although the degradation of NURR1 by the UPS has been reported previously, our study is the first to connect mechanistically this fact with pathology. One study identified Ser^347^ as a site for phosphorylation by AKT that marks this transcription factor for proteasomal degradation. However, the relevance of AKT leading to the degradation of NURR1 is not clear considering that AKT is a survival kinase that should protect NURR1. In fact, AKT phosphorylates GSK-3-α and β at Ser21 and Ser9, respectively, in their pseudosubstrate domain, leading to its inhibition. It is therefore likely that signals that activate AKT will stabilize NURR1 through the inhibition of GSK-3. Another study identified the first 31 N-terminal residues of NURR1 as a target of proteasomal degradation in several cell types [58]. This study was conducted under standard growth conditions and therefore did not provide a direct link with pathology. It is very likely that NURR1, as several other proteins, might have several motifs for UPS targeting. In fact, the Core 2 mutant still incorporates ubiquitin to some extent.

The finding that α-SYN aggregates reduce the dopaminergic phenotype by GSK-3-mediated degradation of NURR1 suggests that GSK-3 inhibitors might be a therapeutic option to preserve the nigrostriatal track in synucleinopathies such as Parkinson’s disease.

## Supplementary Information

Below is the link to the electronic supplementary material.

Supplementary file1 (PDF 754 KB)

## Data Availability

Data will be made available on reasonable request.

## References

[1] Martinat C, Bacci JJ, Leete T, Kim J, Vanti WB, Newman AH, Cha JH, Gether U, Wang H, Abeliovich A (2006). Cooperative transcription activation by Nurr1 and Pitx3 induces embryonic stem cell maturation to the midbrain dopamine neuron phenotype. Proc Natl Acad Sci U S A.

[2] Jankovic J, Chen S, Le WD (2005). The role of Nurr1 in the development of dopaminergic neurons and Parkinson's disease. Prog Neurobiol.

[3] Decressac M, Volakakis N, Bjorklund A, Perlmann T (2013). NURR1 in Parkinson disease–from pathogenesis to therapeutic potential. Nat Rev Neurol.

[4] Kadkhodaei B, Ito T, Joodmardi E, Mattsson B, Rouillard C, Carta M, Muramatsu S, Sumi-Ichinose C, Nomura T, Metzger D, Chambon P, Lindqvist E, Larsson NG, Olson L, Bjorklund A, Ichinose H, Perlmann T (2009). Nurr1 is required for maintenance of maturing and adult midbrain dopamine neurons. J Neurosci.

[5] Sakurada K, Ohshima-Sakurada M, Palmer TD, Gage FH (1999). Nurr1, an orphan nuclear receptor, is a transcriptional activator of endogenous tyrosine hydroxylase in neural progenitor cells derived from the adult brain. Development.

[6] Schimmel JJ, Crews L, Roffler-Tarlov S, Chikaraishi DM (1999). 4.5 kb of the rat tyrosine hydroxylase 5' flanking sequence directs tissue specific expression during development and contains consensus sites for multiple transcription factors. Brain Res Mol Brain Res.

[7] Kim KS, Kim CH, Hwang DY, Seo H, Chung S, Hong SJ, Lim JK, Anderson T, Isacson O (2003). Orphan nuclear receptor Nurr1 directly transactivates the promoter activity of the tyrosine hydroxylase gene in a cell-specific manner. J Neurochem.

[8] Sacchetti P, Mitchell TR, Granneman JG, Bannon MJ (2001). Nurr1 enhances transcription of the human dopamine transporter gene through a novel mechanism. J Neurochem.

[9] Hermanson E, Joseph B, Castro D, Lindqvist E, Aarnisalo P, Wallen A, Benoit G, Hengerer B, Olson L, Perlmann T (2003). Nurr1 regulates dopamine synthesis and storage in MN9D dopamine cells. Exp Cell Res.

[10] Hermanson E, Borgius L, Bergsland M, Joodmardi E, Perlmann T (2006). Neuropilin1 is a direct downstream target of Nurr1 in the developing brain stem. J Neurochem.

[11] Wallen AA, Castro DS, Zetterstrom RH, Karlen M, Olson L, Ericson J, Perlmann T (2001). Orphan nuclear receptor Nurr1 is essential for Ret expression in midbrain dopamine neurons and in the brain stem. Mol Cell Neurosci.

[12] Parkinson GM, Dayas CV, Smith DW (2015). Age-related gene expression changes in substantia nigra dopamine neurons of the rat. Mech Ageing Dev.

[13] Le W, Conneely OM, Zou L, He Y, Saucedo-Cardenas O, Jankovic J, Mosier DR, Appel SH (1999). Selective agenesis of mesencephalic dopaminergic neurons in Nurr1-deficient mice. Exp Neurol.

[14] Zetterstrom RH, Solomin L, Jansson L, Hoffer BJ, Olson L, Perlmann T (1997). Dopamine neuron agenesis in Nurr1-deficient mice. Science.

[15] Xu PY, Liang R, Jankovic J, Hunter C, Zeng YX, Ashizawa T, Lai D, Le WD (2002). Association of homozygous 7048G7049 variant in the intron six of Nurr1 gene with Parkinson's disease. Neurology.

[16] Le WD, Xu P, Jankovic J, Jiang H, Appel SH, Smith RG, Vassilatis DK (2003). Mutations in NR4A2 associated with familial Parkinson disease. Nat Genet.

[17] Gomez-Benito M, Granado N, Garcia-Sanz P, Michel A, Dumoulin M, Moratalla R (2020). Modeling Parkinson's disease with the alpha-synuclein protein. Front Pharmacol.

[18] Olgiati S, Thomas A, Quadri M, Breedveld GJ, Graafland J, Eussen H, Douben H, de Klein A, Onofrj M, Bonifati V (2015). Early-onset parkinsonism caused by alpha-synuclein gene triplication: Clinical and genetic findings in a novel family. Parkinsonism Relat Disord.

[19] Ahn TB, Kim SY, Kim JY, Park SS, Lee DS, Min HJ, Kim YK, Kim SE, Kim JM, Kim HJ, Cho J, Jeon BS (2008). alpha-Synuclein gene duplication is present in sporadic Parkinson disease. Neurology.

[20] Fujiwara H, Hasegawa M, Dohmae N, Kawashima A, Masliah E, Goldberg MS, Shen J, Takio K, Iwatsubo T (2002). alpha-Synuclein is phosphorylated in synucleinopathy lesions. Nat Cell Biol.

[21] Anderson JP, Walker DE, Goldstein JM, de Laat R, Banducci K, Caccavello RJ, Barbour R, Huang J, Kling K, Lee M, Diep L, Keim PS, Shen X, Chataway T, Schlossmacher MG, Seubert P, Schenk D, Sinha S, Gai WP, Chilcote TJ (2006). Phosphorylation of Ser-129 is the dominant pathological modification of alpha-synuclein in familial and sporadic Lewy body disease. J Biol Chem.

[22] Lin X, Parisiadou L, Sgobio C, Liu G, Yu J, Sun L, Shim H, Gu XL, Luo J, Long CX, Ding J, Mateo Y, Sullivan PH, Wu LG, Goldstein DS, Lovinger D, Cai H (2012). Conditional expression of Parkinson's disease-related mutant alpha-synuclein in the midbrain dopaminergic neurons causes progressive neurodegeneration and degradation of transcription factor nuclear receptor related 1. J Neurosci.

[23] Xu C, Kim NG, Gumbiner BM (2009). Regulation of protein stability by GSK3 mediated phosphorylation. Cell Cycle.

[24] Kim S, Lim J, Bang Y, Moon J, Kwon MS, Hong JT, Jeon J, Seo H, Choi HJ (2018). Alpha-synuclein suppresses retinoic acid-induced neuronal differentiation by targeting the glycogen synthase kinase-3beta/beta-catenin signaling pathway. Mol Neurobiol.

[25] Majd S, Power JH, Grantham HJ (2015). Neuronal response in Alzheimer's and Parkinson's disease: the effect of toxic proteins on intracellular pathways. BMC Neurosci.

[26] Gassowska M, Czapski GA, Pajak B, Cieslik M, Lenkiewicz AM, Adamczyk A (2014). Extracellular alpha-synuclein leads to microtubule destabilization via GSK-3beta-dependent Tau phosphorylation in PC12 cells. PLoS ONE.

[27] Kawakami F, Suzuki M, Shimada N, Kagiya G, Ohta E, Tamura K, Maruyama H, Ichikawa T (2011). Stimulatory effect of alpha-synuclein on the tau-phosphorylation by GSK-3beta. FEBS J.

[28] Duka T, Duka V, Joyce JN, Sidhu A (2009). Alpha-Synuclein contributes to GSK-3beta-catalyzed Tau phosphorylation in Parkinson's disease models. FASEB J.

[29] Hur EM, Zhou FQ (2010). GSK3 signalling in neural development. Nat Rev Neurosci.

[30] Phiel CJ, Wilson CA, Lee VM, Klein PS (2003). GSK-3alpha regulates production of Alzheimer's disease amyloid-beta peptides. Nature.

[31] Himmelstein DS, Ward SM, Lancia JK, Patterson KR, Binder LI (2012). Tau as a therapeutic target in neurodegenerative disease. Pharmacol Ther.

[32] Mines MA, Beurel E, Jope RS (2011). Regulation of cell survival mechanisms in Alzheimer's disease by glycogen synthase kinase-3. Int J Alzheimers Dis.

[33] Credle JJ, George JL, Wills J, Duka V, Shah K, Lee YC, Rodriguez O, Simkins T, Winter M, Moechars D, Steckler T, Goudreau J, Finkelstein DI, Sidhu A (2015). GSK-3beta dysregulation contributes to parkinson's-like pathophysiology with associated region-specific phosphorylation and accumulation of tau and alpha-synuclein. Cell Death Differ.

[34] Kwok JB, Hallupp M, Loy CT, Chan DK, Woo J, Mellick GD, Buchanan DD, Silburn PA, Halliday GM, Schofield PR (2005). GSK3B polymorphisms alter transcription and splicing in Parkinson's disease. Ann Neurol.

[35] Golpich M, Amini E, Hemmati F, Ibrahim NM, Rahmani B, Mohamed Z, Raymond AA, Dargahi L, Ghasemi R, Ahmadiani A (2015). Glycogen synthase kinase-3 beta (GSK-3beta) signaling: Implications for Parkinson's disease. Pharmacol Res.

[36] Li DW, Liu ZQ, Chen W, Yao M, Li GR (2014). Association of glycogen synthase kinase-3beta with Parkinson's disease (review). Mol Med Rep.

[37] Emmanouilidou E, Melachroinou K, Roumeliotis T, Garbis SD, Ntzouni M, Margaritis LH, Stefanis L, Vekrellis K (2010). Cell-produced alpha-synuclein is secreted in a calcium-dependent manner by exosomes and impacts neuronal survival. J Neurosci.

[38] Pantazopoulou M, Brembati V, Kanellidi A, Bousset L, Melki R, Stefanis L (2020). Distinct alpha-Synuclein species induced by seeding are selectively cleared by the Lysosome or the Proteasome in neuronally differentiated SH-SY5Y cells. J Neurochem.

[39] Garcia-Yague AJ, Rada P, Rojo AI, Lastres-Becker I, Cuadrado A (2013). Nuclear import and export signals control the subcellular localization of Nurr1 protein in response to oxidative stress. J Biol Chem.

[40] Escoll M, Lastra D, Pajares M, Robledinos-Anton N, Rojo AI, Fernandez-Gines R, Mendiola M, Martinez-Marin V, Esteban I, Lopez-Larrubia P, Gargini R, Cuadrado A (2020). Transcription factor NRF2 uses the Hippo pathway effector TAZ to induce tumorigenesis in glioblastomas. Redox Biol.

[41] Patterson JR, Polinski NK, Duffy MF, Kemp CJ, Luk KC, Volpicelli-Daley LA, Kanaan NM, Sortwell CE (2019) Generation of alpha-synuclein preformed fibrils from monomers and use in vivo. J Vis Exp (148). doi:10.3791/59758 10.3791/59758 PMC10337163 31205308

[42] Volpicelli-Daley LA, Luk KC, Lee VM (2014). Addition of exogenous alpha-synuclein preformed fibrils to primary neuronal cultures to seed recruitment of endogenous alpha-synuclein to Lewy body and Lewy neurite-like aggregates. Nat Protoc.

[43] Pajares M, Jimenez-Moreno N, Garcia-Yague AJ, Escoll M, de Ceballos ML, Van Leuven F, Rabano A, Yamamoto M, Rojo AI, Cuadrado A (2016). Transcription factor NFE2L2/NRF2 is a regulator of macroautophagy genes. Autophagy.

[44] Rojo AI, Innamorato NG, Martin-Moreno AM, De Ceballos ML, Yamamoto M, Cuadrado A (2010). Nrf2 regulates microglial dynamics and neuroinflammation in experimental Parkinson's disease. Glia.

[45] Lastres-Becker I, Ulusoy A, Innamorato NG, Sahin G, Rabano A, Kirik D, Cuadrado A (2012). alpha-Synuclein expression and Nrf2 deficiency cooperate to aggravate protein aggregation, neuronal death and inflammation in early-stage Parkinson's disease. Hum Mol Genet.

[46] Vekrellis K, Xilouri M, Emmanouilidou E, Stefanis L (2009). Inducible over-expression of wild type alpha-synuclein in human neuronal cells leads to caspase-dependent non-apoptotic death. J Neurochem.

[47] Fang X, Yu SX, Lu Y, Bast RC, Woodgett JR, Mills GB (2000). Phosphorylation and inactivation of glycogen synthase kinase 3 by protein kinase A. Proc Natl Acad Sci U S A.

[48] Sutherland C, Leighton IA, Cohen P (1993). Inactivation of glycogen synthase kinase-3 beta by phosphorylation: new kinase connections in insulin and growth-factor signalling. Biochem J.

[49] Shaw G, Morse S, Ararat M, Graham FL (2002). Preferential transformation of human neuronal cells by human adenoviruses and the origin of HEK 293 cells. FASEB J.

[50] Lin YC, Boone M, Meuris L, Lemmens I, Van Roy N, Soete A, Reumers J, Moisse M, Plaisance S, Drmanac R, Chen J, Speleman F, Lambrechts D, Van de Peer Y, Tavernier J, Callewaert N (2014). Genome dynamics of the human embryonic kidney 293 lineage in response to cell biology manipulations. Nat Commun.

[51] Shaw M, Cohen P, Alessi DR (1997). Further evidence that the inhibition of glycogen synthase kinase-3beta by IGF-1 is mediated by PDK1/PKB-induced phosphorylation of Ser-9 and not by dephosphorylation of Tyr-216. FEBS Lett.

[52] Bhat RV, Shanley J, Correll MP, Fieles WE, Keith RA, Scott CW, Lee CM (2000). Regulation and localization of tyrosine216 phosphorylation of glycogen synthase kinase-3beta in cellular and animal models of neuronal degeneration. Proc Natl Acad Sci U S A.

[53] Wang W, Yang Y, Ying C, Li W, Ruan H, Zhu X, You Y, Han Y, Chen R, Wang Y, Li M (2007). Inhibition of glycogen synthase kinase-3beta protects dopaminergic neurons from MPTP toxicity. Neuropharmacology.

[54] Zhao Q, Ye J, Wei N, Fong C, Dong X (2016). Protection against MPP(+)-induced neurotoxicity in SH-SY5Y cells by tormentic acid via the activation of PI3-K/Akt/GSK3beta pathway. Neurochem Int.

[55] Chen G, Bower KA, Ma C, Fang S, Thiele CJ, Luo J (2004). Glycogen synthase kinase 3beta (GSK3beta) mediates 6-hydroxydopamine-induced neuronal death. FASEB J.

[56] Wang HM, Zhang T, Li Q, Huang JK, Chen RF, Sun XJ (2013). Inhibition of glycogen synthase kinase-3beta by lithium chloride suppresses 6-hydroxydopamine-induced inflammatory response in primary cultured astrocytes. Neurochem Int.

[57] Xie CL, Lin JY, Wang MH, Zhang Y, Zhang SF, Wang XJ, Liu ZG (2016). Inhibition of Glycogen Synthase Kinase-3beta (GSK-3beta) as potent therapeutic strategy to ameliorates L-dopa-induced dyskinesia in 6-OHDA parkinsonian rats. Sci Rep.

[58] Alvarez-Castelao B, Losada F, Ahicart P, Castano JG (2013). The N-terminal region of Nurr1 (a.a 1-31) is essential for its efficient degradation by the ubiquitin proteasome pathway. PLoS One.

[59] Jo AY, Kim MY, Lee HS, Rhee YH, Lee JE, Baek KH, Park CH, Koh HC, Shin I, Lee YS, Lee SH (2009). Generation of dopamine neurons with improved cell survival and phenotype maintenance using a degradation-resistant nurr1 mutant. Stem Cells.

[60] Robertson H, Hayes JD, Sutherland C (2018). A partnership with the proteasome; the destructive nature of GSK3. Biochem Pharmacol.

[61] Garwood ER, Bekele W, McCulloch CE, Christine CW (2006). Amphetamine exposure is elevated in Parkinson's disease. Neurotoxicology.

[62] Christine CW, Garwood ER, Schrock LE, Austin DE, McCulloch CE (2010). Parkinsonism in patients with a history of amphetamine exposure. Mov Disord.

[63] Meredith GE, Rademacher DJ (2011). MPTP mouse models of Parkinson's disease: an update. J Parkinsons Dis.

[64] Doudet D, Gross C, Lebrun-Grandie P, Bioulac B (1985). MPTP primate model of Parkinson's disease: a mechanographic and electromyographic study. Brain Res.

[65] Stefanis L (2012). alpha-Synuclein in Parkinson's disease. Cold Spring Harb Perspect Med.

[66] Innamorato NG, Jazwa A, Rojo AI, Garcia C, Fernandez-Ruiz J, Grochot-Przeczek A, Stachurska A, Jozkowicz A, Dulak J, Cuadrado A (2010). Different susceptibility to the Parkinson's toxin MPTP in mice lacking the redox master regulator Nrf2 or its target gene heme oxygenase-1. PLoS ONE.

[67] Granado N, Lastres-Becker I, Ares-Santos S, Oliva I, Martin E, Cuadrado A, Moratalla R (2011). Nrf2 deficiency potentiates methamphetamine-induced dopaminergic axonal damage and gliosis in the striatum. Glia.

[68] Baptista MJ, O'Farrell C, Daya S, Ahmad R, Miller DW, Hardy J, Farrer MJ, Cookson MR (2003). Co-ordinate transcriptional regulation of dopamine synthesis genes by alpha-synuclein in human neuroblastoma cell lines. J Neurochem.

[69] Decressac M, Kadkhodaei B, Mattsson B, Laguna A, Perlmann T, Bjorklund A (2012). alpha-Synuclein-induced down-regulation of Nurr1 disrupts GDNF signaling in nigral dopamine neurons. Sci Transl Med.

[70] Chu Y, Muller S, Tavares A, Barret O, Alagille D, Seibyl J, Tamagnan G, Marek K, Luk KC, Trojanowski JQ, Lee VMY, Kordower JH (2019). Intrastriatal alpha-synuclein fibrils in monkeys: spreading, imaging and neuropathological changes. Brain.

[71] Chu Y, Kordower JH (2021). GDNF signaling in subjects with minimal motor deficits and Parkinson's disease. Neurobiol Dis.

[72] Decressac M, Ulusoy A, Mattsson B, Georgievska B, Romero-Ramos M, Kirik D, Bjorklund A (2011). GDNF fails to exert neuroprotection in a rat alpha-synuclein model of Parkinson's disease. Brain.

[73] Jia C, Qi H, Cheng C, Wu X, Yang Z, Cai H, Chen S, Le W (2020). alpha-Synuclein negatively regulates Nurr1 expression through NF-kappaB-related mechanism. Front Mol Neurosci.

[74] Devine MJ (2012). Proteasomal inhibition as a treatment strategy for Parkinson's disease: the impact of alpha-synuclein on Nurr1. J Neurosci.

[75] Wingate AD, Campbell DG, Peggie M, Arthur JS (2006). Nur77 is phosphorylated in cells by RSK in response to mitogenic stimulation. Biochem J.

[76] Zhang T, Jia N, Fei E, Wang P, Liao Z, Ding L, Yan M, Nukina N, Zhou J, Wang G (2007). Nurr1 is phosphorylated by ERK2 in vitro and its phosphorylation upregulates tyrosine hydroxylase expression in SH-SY5Y cells. Neurosci Lett.

[77] Kuure S, Popsueva A, Jakobson M, Sainio K, Sariola H (2007). Glycogen synthase kinase-3 inactivation and stabilization of beta-catenin induce nephron differentiation in isolated mouse and rat kidney mesenchymes. J Am Soc Nephrol.

