# Supplementary material for: α-Synuclein Induces the GSK-3-Mediated Phosphorylation and Degradation of NURR1 and Loss of Dopaminergic Hallmarks
Source: Mol Neurobiol. 2021 Oct 5;58(12):6697–711. doi: 10.1007/s12035-021-02558-9 (PMC8639559; doi:10.1007/s12035-021-02558-9)
Supplement: Supplementary file 1 — Supplementary file1 (PDF 754 KB) [file 12035_2021_2558_MOESM1_ESM.pdf]

α-Synuclein induces the GSK-3 mediated phosphorylation and degradation of NURR1 and loss of dopaminergic hallmarks

Ángel Juan García-Yagüe[1], Isabel Lastres-Becker[1], Leonidas Stefanis 2, Demetrios K. Vassilatis [3] and Antonio Cuadrado[1]*

*

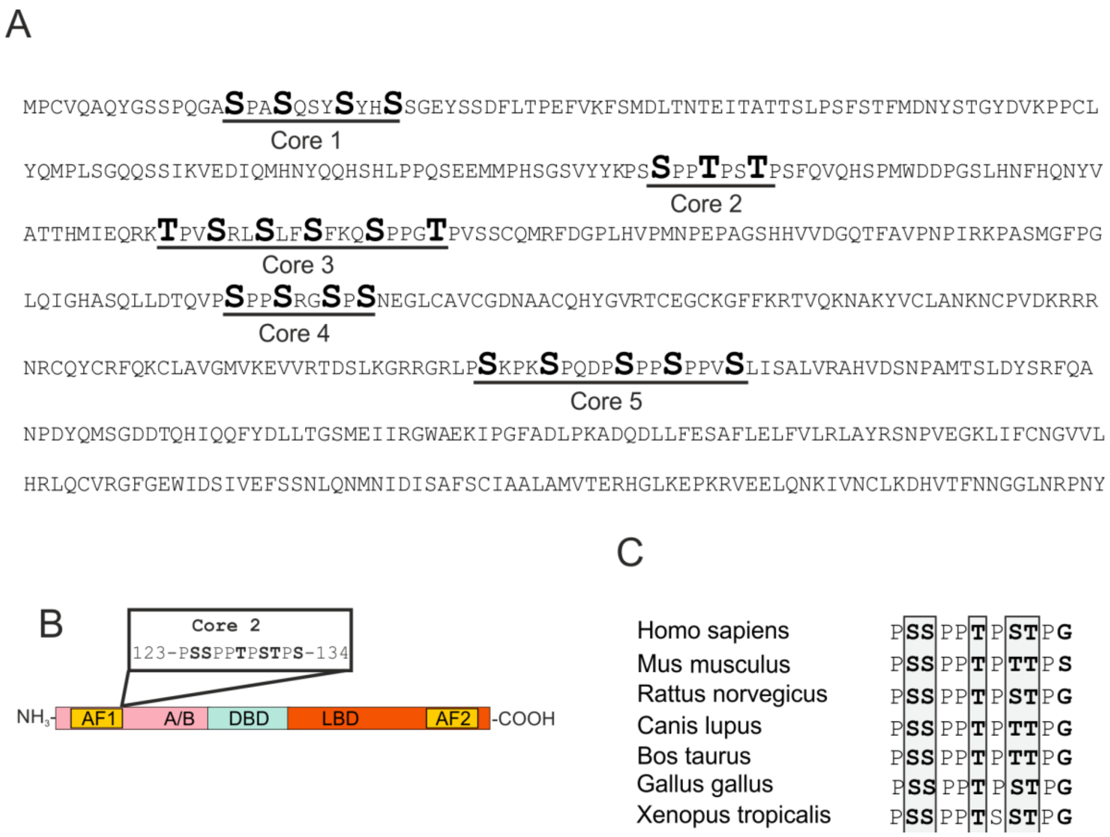

Supplemental figure 1. Predictions analysis the GSK-3 phosphorylation site in NURR1. **A**, NURR1 primary sequence. Putative serines or threonines that according NetPhos 2.0 might be phosphorylated by GSK-3 are marked in bold. These residues are grouped at five clusters that we called Core 1 to 5. B, schematic localization of Core 2 in NURR1. AF1, activation function-1; A/B, ligand-independent domains A and B; DBD, DNA binding domain ; LBD, ligand binding domain. **C**, sequence comparison of Core 2 in vertebrates, indicating a high degree of evolutionary conservation.

Supplemental Materials and Methods

Plasmids. The vectors pcDNA3.1-Nurr1^{MUT2}-V5/6xHis mutants were generated with the Gene Tailor site-directed mutagenesis system (Invitrogen) using as template pcDNA3.1-Nurr1^{WT}-V5His. Oligonucleotides to generate Nurr1^{MUT2} mutant are described in Table 1. Lentiviral plasmid pWPXL-Nurr1^{WT}-V5/6xHis and pWPXL-Nurr1^{MUT2}-V5/6xHis construction had been performed using Nurr1^{WT} and Nurr1^{MUT2} fragment from original plasmids pcDNA3.1-Nurr1^{WT}-V5/6xHis and pcDNA3.1-Nurr1^{MUT2}-V5/6xHis. Both plasmids were used as a template to generate Nurr1PCRinsert with oligonucleotides that introduce SpeI restriction sites at both ends and EcoRI check insert orientation (Table 1. The expression construct pEGFP-Nurr1^{WT}-V5/6xHis full length and its NH_3-terminal delection mutants was created using as template pcDNA3.1-Nurr1^{WT}-V5/6xHis to generate Nurr1PCRinsert with oligonucleotides that introduce HindIII and BglII restriction site at both end. Oligonucleotides to generate Nurr1 delection mutant are described in Table 1. pEGFP-C1 was using as transfection control in the experiment. The coding region of the human NURR1 cDNA was amino-terminally epitope-tagged by ligating it the into pCMV-Myc expression vector (Clontech). The purified pCMV-Myc-NURR1 expression vector was transiently transfected into SHSY-5Y cells using Lipofectamine 2000 Transfection Reagent (Invitrogen) using the manufacturer's instructions. Cell protein lysates were prepared 36 h after transfection and analyzed by immunoblot.

α-Synuclein Pre-Formed Fibrils (PFF). Purified α- SYN monomers for PFF fibrilization was diluted to 5 mg/mL with final buffer composition of 100 mM NaCl, 10 mM Phosphate, pH 7.2–7.6 and constantly agitated at 1000rpm, 37°C for 7 days. Fibril formation was confirmed using sedimentation followed by gel electrophoresis (Suppl.

Mat Fig 1). Sedimentation was performed by centrifugation at 100,000 xg for 30 min. Samples were loaded on 15% Acri-Bis gel and ran at 40 mA for 1h. Gels were stained with coomassie brilliant blue (Sima-Aldrich). α- SYN PFFs were aliquoted and stored in −80°C until use. For SH- SY5Y cell treatment with PFFs, cells were seeded for mRNA analyses in 6- well plates (~5 x 10^4 cells/ well) and for protein in 60-mm plates (~5 x 10^4 cells/plate). Cells were seeded (~5 x 10^3 cells/ well) on coverslips for immunocytochemistry in 24-well plates. α- SYN PFFs were thawed at 25°C, diluted to 0.1 mg/mL with sterile DPBS, and sonicated with 60 pulses at 10% power (total of 30 sec, 0.5 sec on, 0.5 sec off). Sonicated PFFs were diluted with cell culture media then added to 2µg/ml on 6- well plates and 60-mm plates or 1µg/ml on 24-well plates. The sonication step is expected to produce a heterogeneous population of fibrils ranging in length from 20 to 100 nm.

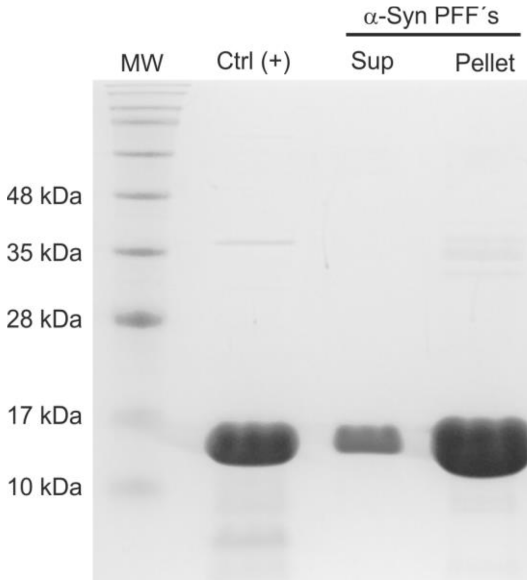

Quality control of PFF production by sedimentation assay. PFFs were prepared as described above. The supernatant and pellet were resolved by SDS-PAGE and stained with Coomassie Blue.

Lentiviral vector production and infection. The lentivirus particles used in this study, including, shGSK-3α (NM_019884, TRCN0000038682), shGSK-3β (NM_002093, TRCN0000039564), Nurr1^{WT}, Nurr1^{MUT2}, vector pWXL control and

scrambled RNA vector (shCtrl 1864, Addgene) were generated in HEK 293 T cells. Briefly, a mixture of 6 µg of envelope plasmid (pMD2G 12259, Addgene), 6 µg of packaging plasmid (psPAX2 12260, Addgene) and 10 µg of transfer vector was prepared in DMEM media (Sigma-Aldrich) and transfected into HEK293T cells (2×10^6 cells/100 mm dishes) using TransFectin Lipid Reagent (Bio-RAD) and cultured for 24 h. Lentivirus containing supernatant was harvested and passed through a 0.45 µm filter. Cells were infected in the presence of 4 µg/ml polybrene (Sigma-Aldrich) and selected with 1 µg/ml puromycin (Sigma-Aldrich).

Ubiquitination assay. Cells were then lysed in a RIPA buffer (150mM NaCl, 25mM Tris-HCl, pH 7.5, 1% Nonidet P-40, 1% sodium deoxycholate, 1% Triton-X100, 0.1% SDS, 1 mM phenylmethylsulfonyl fluoride, 1mMNaF, 1mMsodium pyrophosphate, 1mMsodium orthovanadate, 1 g/ml leupeptin). After that, the samples were kept for 30 min at 4°C in a rotating wheel and centrifuged at 13000 rpm for 10min. Three microliters of the anti-V5 (Invitrogen) were added per lysate, and after incubation for 2 h at 4°C in a rotating wheel, gamma-bind Sepharose-protein G was added (Amersham Biosciences), followed by incubation for 1 h at 4°C. The complexes were harvested by centrifugation, washed three washes with RIPA buffer, immunoprecipitated complexes were eluted in sample buffer by boiling, electrophoresed through SDS–polyacrylamide gels, and subjected to immunoblot analysis. Mouse IgG TrueBlot (eBiosciences) was used as a peroxidase-conjugated secondary antibody (1:10,000 dilution) because it reduces interference by the 55-kDa heavy and 23-kDa light chains of the immunoprecipitation antibody

2D PAGE (**Two-dimensional electrophoresis). A**fter 24 h of recovery from transfection, the cells were resuspended in 200 µl 2D lysis buffer (7 M urea, 2 M thiourea,4% (w/v) Chaps, 30 mM Tris–HCl, pH 7.5). For 2D-gel electrophoresis, 50 µl

of sample was dissolved in 300 µl of 2D rehydration buffer (7 M urea, 2 M thiourea, 4% (w/v) Chaps, 50 mM dithiothreitol, and 1% (v/v) IPG 4–7 buffer). Samples were loaded on immobilized pH gradient Immobiline DryStrip gels 4–7 (GE Healthcare) and isoelectrofocused in an IPGphor3 basic unit (GE Healthcare) by applying a total of 55 kV/h (for each experiment, samples are processed in the same run of isoelectrofocalization). Then, the strips were first equilibrated for 10 min with 2% (w/v) DTT, 50 mM Tris–HCl, pH 8.8, 30% glycerol, 5% SDS, 0.05% bromophenol blue and then with 4% (w/v) iodoacetamide, 50 mM Tris–HCl, pH 8.8, 30% glycerol, 5% SDS, and 0.05% bromophenol blue. The equilibrated strips were layered onto a 12% SDS–PAGE gel to perform the second-dimension separation. After blotting to Immobilon-P membranes, immunoblots were performed as described above, using anti-V5 primary antibody and peroxidase conjugated anti-mouse as second antibody. All of the 2D reagents mentioned above were supplied by PlusOne (GE Healthcare).

<u>Supplemental Table 1.</u> Primers used for plasmids construction.

Plasmid	Forward primer (5’-3’)	Reverse primer (5’-3’)
pcDNA3.1-Nurr1^{MUT2}-V5/6xHis	GGTCGGTTTACTACAAGCCCGCTGCGCCCCCGGCACCCGCCGCCCCGGCCTTCCAGGTG	GGGCTTGTAGTAAACCGACCCGCTGTGTGG
pWPXL-Nurr1^{WT}-V5/6xHis	TGCAACTAGTATGCCTTGTGTTCAGGCGCAGT	GTACACTAGTTCAATGGTGATGGTGATGAT
pWPXL-Nurr1^{MUT2}-V5/6xHis		
pEGFP- Nurr1^{WT}-V5/6xHis	AGTCAGATCTATGCCTTGTGTTCAGGCGCAGTA	GCATAAGCTTGAGGCTGATCAGCGGGTTTAAACTCAATG
pEGFP- Nurr1 Δ1-V5/6xHis	AGTCAGATCTTTTAGCATGGACCTCACCAACACTGAAAT	
pEGFP- Nurr1 Δ2-V5/6xHis	AGTCAGATCTAAGACACCTGTCTCCCGCCTGTCACTC	
pEGFP- Nurr1 Δ3-V5/6xHis	AGTCAGATCTTTCGACGGGCCTCTGCACGTCCCCATGAA	
pEGFP- Nurr1 Δ4-V5/6xHis	AGTCAGATCTACTTGTGAGGGCTGCAAAGGTTTC	
pEGFP- Nurr1 Δ5-V5/6xHis	AGTCAGATCTGCCCACGTCGATTCCAATCCGGCAATGAC	

Supplemental Table 2. Antibodies.

Antibody	Source	Catalog. number	Applications
V5	Invitrogen	R960-25	WB 1:2000
HA	Covance	MMS-101R	WB 1:2000
GFP	Sigma-Aldrich	G1546	WB 1:4000
GAPDH	Merck-Millipore	CB1001	WB 1:15000
NURR1	Santa Cruz Biotechlogy	sc-81345	WB 1:1000
β-actin	Santa Cruz Biotechlogy	sc-1616	WB 1:4000
Lamin B	Santa Cruz Biotechlogy	sc-6217	WB 1:2000
β-catenin	Becton Dickinson	610153	WB 1:2000
AKT-pSer^{473}	Cell Signaling Technology	4058	WB 1:2000
AKT	Becton Dickinson	610860	WB 1:1000
GSK-3β-pSer^{9}	Cell Signaling Technology	9336	WB 1:2000
GSK-3β	Becton Dickinson	610201	WB 1:2000
RET	Cell Signaling Technology	3223	WB 1:1000
α-SYN	BD transduction laboratories	610787	WB: 1000<br>IF 1:100
α-SYN-pSer^{129}	Abcam	Ab59264	WB 1:1000<br>IF 1:100
GSK-3α/β	Santa Cruz Biotechlogy	sc-721	WB 1:1000
TH	Merck-Millipore	AB152	WB 1:2000

Supplemental Table 3. List of primers used for qRT-PCR.

**Gene**	**Forward primer (5’-3`)**	**Reverse primer (5’-3’)**	**Species**
NURR1	ACCACTCTTCGGGAGAATACA	GGCATTTGGTACAAGCAAGGT	Human
TH	GTCCCCGCGGTTCATTGG	CCTTCTCCTCAAAGGCCACA	
RET	TGATGGCACTAACACTGGGT	CCGCTGAGGGTGAAAGCAT	
ACTB	TCCTTCCTGGGCATGGAG	AGGAGGAGCAATGATCTTGATCTT	
GAPDH	CTCTCTGCTCCTCCTGTTCGAC	TGAGCGATGTGGCTCGGCT	
